# Supplementary material for: EPHA3 Could Be a Novel Prognosis Biomarker and Correlates with Immune Infiltrates in Bladder Cancer
Source: Cancers (Basel). 2023 Jan 19;15(3):621. doi: 10.3390/cancers15030621 (PMC9913674; doi:10.3390/cancers15030621)
Supplement: Supplementary file 1 [file cancers-15-00621-s001.zip › cancers-2096656-File S1.pdf]

cancers

MDPI

Supplementary Materials

EPHA3 Could Be a Novel Prognosis Biomarker and Correlates with Immune Infiltrates in Bladder Cancer

Junpeng Liu, Zewen Zhou, Yifan Jiang, Yuzhao Lin, Yunzhi Yang, Chongjiang Tian, Jinwen Liu, Hao Lin and Bin Huang

1. The specific sequence of gene EPHA3:

GCTAAATTCTGGCCGTTTTTGGCTTTTTTGTTAGACGAAGCTTGGGCTG-
CAGGTCGACTCTA-
GAGGATCCCGCCACCATGGATTGTCAGCTCTCCATCCTCCTCCTTCTCAGCTGCT
CTGTTCTCGACAGCTTCGGGGAACTGATTCCG-
CAGCCTTCCAATGAAGTCAATCTACTGGAT-
TCAAAAACAATTCAAGGGGAGCTGGGCTGGATCTCTTATCCATCACATGGGTGG
GAAGAGATCAGTGGTGTGGATGAACATTACACACCCATCAGGACTTAC-
CAGGTGTG-
CAATGTCATGGACCACAGTCAAAACAATTGGCTGAGAACAAACTGGGTCCCCA
GGAACTCAGCTCAGAAGATTTATGTGGAGCTCAAGTTCACTCTACGAGACTG-
CAA-
TAGCATTCCATTGGTTTTAGGAACTTGCAAGGAGACATTCAACCTGTACTACATG
GAGTCTGATGATGATCATGGGGTGAAATTTCGAGAGCATCAGTTTACAAA-
GATTGACAC-
CATTGCAGCTGATGAAAGTTTCACTCAAATGGATCTTGGGGACCGTATTCTGAAG
CTCAACACTGAGATTAGAGAAGTAGGTCCTGTCAACAAGAAGGGATTTTATTT-
GG-
CATTTCAAGATGTTGGTGCTTGTGTTGCCTTGGTGTCTGTGAGAGTATACTTCAAA
AAGTGCCCATTTACAGTGAAGAATCTGGCTATGTTTCCAGACACGG-
TACCCATGGACTCCCAGTCCCTGGTGGAGGTTAGAGGGTCTTGTGTCAACAATTC
TAAGGAGGAAGATCCTCCAAGGATGTACTGCAGTACAGAAGGCGAATGGCTT-
GTACCCATT-
GGCAAGTGTTCCTGCAATGCTGGCTATGAAGAAAGAGGTTTTATGTGCCAAGCTT
GTCGACCAGGTTTCTACAAGGCATTGGATGGTAA-
TATGAAGTGTGCTAAGTGCCCGCCTCACAGTTCTACTCAGGAAGATGGTTCAATG
AACTGCAGGTGTGAGAATAATTACTTCCGGGCAGACAAA-
GACCCTCCATCCATGGCTT-
GTACCCGACCTCCATCTTCACCAAGAAATGTTATCTCTAATATAAACGAGACCTC
AGTTATCCTGGACTGGAGTTGGCCCCTGGACACAGGAGGCCGGAAA-
GATGTTAC-
CTTCAACATCATATGTAAAAAATGTGGGTGGAATATAAAACAGTGTGAGCCATG
CAGCCCAAATGTCCGCTTCCTCCCTCGACAGTTTGGACTCACCAACACCAC-
GGTGACAG-
TGACAGACCTTCTGGCACATACTAACTACACCTTTGAGATTGATGCCGTTAATGG
GGTGTCAGAGCTGAGCTCCCCACCAAGACAGTTTGCTGCGGTCAG-
CATCACAACTAATCAGGCTGCTCCATCACCTGTCCTGACGATTAAGAAAGATCG
GACCTCCAGAAATAGCATCTCTTTGTCCTGGCAAGAACCTGAACATCCTAATGG-
GATCATATTGGACTACGAGGTCAAATACTATGAAAAGCAGGAACAAGAAACAA
GTTATACCATTCTGAGGGCAAGAGGCACAAATGTTACCATCAGTAGCCTCAA-
GCCTGACAC-
TATATACGTATTCCAAATCCGAGCCCGAACAGCCGCTGGATATGGGACGAACAG

CCGCAAGTTTGAGTTTGAAACTAG-
TCCAGACTCTTTCTCCATCTCTGGTGAAAGTAGCCAAGTGGTCATGATCGCCATT
TCAGCGGCAGTAGCAATTATTCTCCTCACTGTTGTCATCTATGTTTTGATTGG-
GAGGTTCTGTGGCTATAAGTCAAAACATGGGGCAGATGAAAAAAGACTTCATTT
TGGCAATGGGCATTTAAAACTTCCAGGTCTCAGGACTTATGTTGACCCACATA-
CATATGAA-
GACCCTACCCAAGCTGTTCATGAGTTTGCCAAGGAATTGGATGCCACCAACATA
TCCATTGATAAAGTTGTTGGAGCAGGTGAATTTGGAGAGGTGTGCAG-
TGGTCGCTTAAAACTTCCTTCAAAAAAAGAGATTTCAGTGGCCATTAAGACCCT
GAAAGTTGGCTACACAGAAAAGCAGAGGAGAGACTTCCTGGGAGAAGCAA-
GCATTATGG-
GACAGTTTGACCACCCCAATATCATTCGACTGGAAGGAGTTGTTACCAAAAGTA
AGCCAGTTATGATTGTCACAGAATACATGGAGAATGGTTCCTTGGATAGTTTCC-
TAC-
GTAAACACGATGCCCAGTTTACTGTCATTCAGCTAGTGGGGATGCTTCGAGGGA
TAGCATCTGGCATGAAGTACCTGTCAGACATGGGCTATGTTCACCGAGAC-
CTCGCTGCTCG-
GAACATCTTGATCAACAGTAACTTGGTGTGTAAGGTTTCTGATTTCGGACTTTCG
CGTGTCCTGGAGGATGACCCAGAAGCTGCTTATACAACAAGAGGAGGGAA-
GATCCCAATCAGGTGGACATCACCAGAAGCTATAGCCTACCGCAAGTTCACGTC
AGCCAGCGATGTATGGAGTTATGGGATTGTTCTCTGG-
GAGGTGATGTCTTATGGAGAGAGAC-
CATACTGGGAGATGTCCAATCAGGATGTAATTAAAGCTGTAGATGAGGGCTATC
GACTGCCACCCCCCATGGACTGCCCAGCTGCCTTGTATCAGCTGATGCTG-
GACTGCTGG-
CAGAAAGACAGGAACAACAGACCCAAGTTTGAGCAGATTGTTAGTATTCTGGA
CAAGCTTATCCGGAATCCCGGCAGCCTGAAGATCATCACCAGTGCAGCCG-
CAAGGCCATCAAACCTTCTTCTGGACCAAAGCAATGTGGATATCACTACCTTCC
GCACAACAGGTGACTGGCTTAATGGTGTCTGGACAGCACACTG-
CAAGGAAATCTTCAC-
GGGTGTGGAGTACAGTTCTTGTGACACAATAGCCAAGATTTCCACAGATGACAT
GAAAAAGGTTGGTGTCACCGTGGTTGGGCCACAGAAGAAGATCATCAGTAG-
CATTAAA-
GCTCTAGAAACGCAATCAAAGAATGGCCCAGTTCCCGTGGGTATGGACTACAAG
GATGACGATGACAAGGATTACAAAGACGACGATGATAAGGAC-
TATAAGGATGATGAC-
GACAAATGAGCTAGCACATAACTTACGGTAAATGGCCCGCCTGGCTGACCGCCC
AACG

2. Uncropped original Western Blotting images

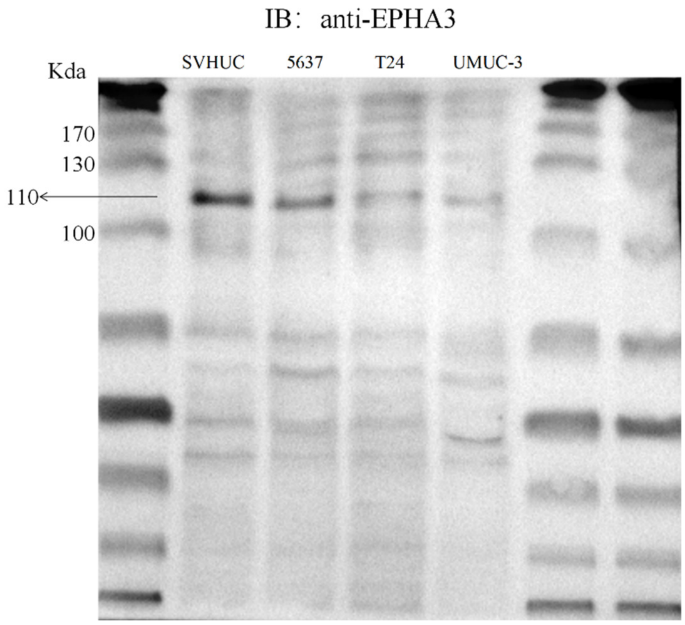

Figure S1. EPHA3.

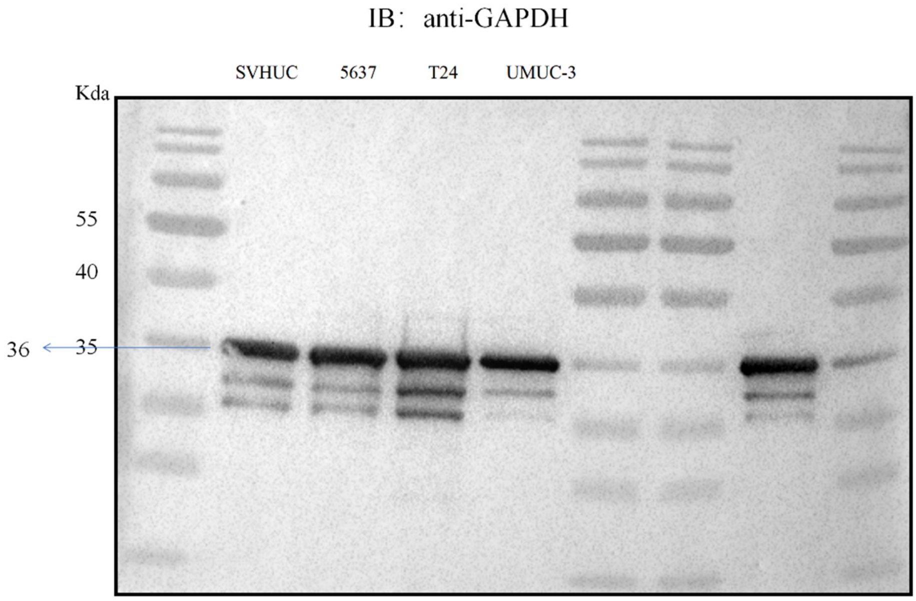

Figure S2. GAPDH.

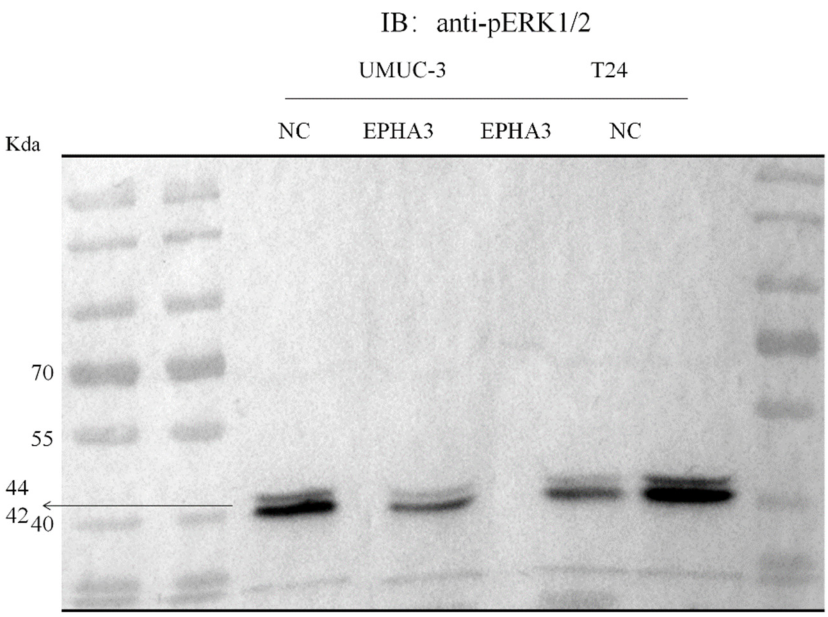

Figure S3. pERK1/2.

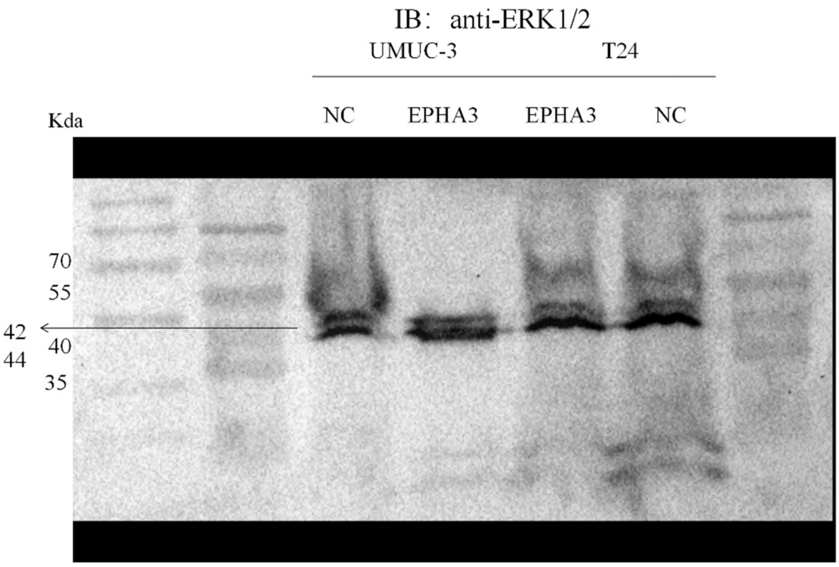

Figure S4. ERK1/2.

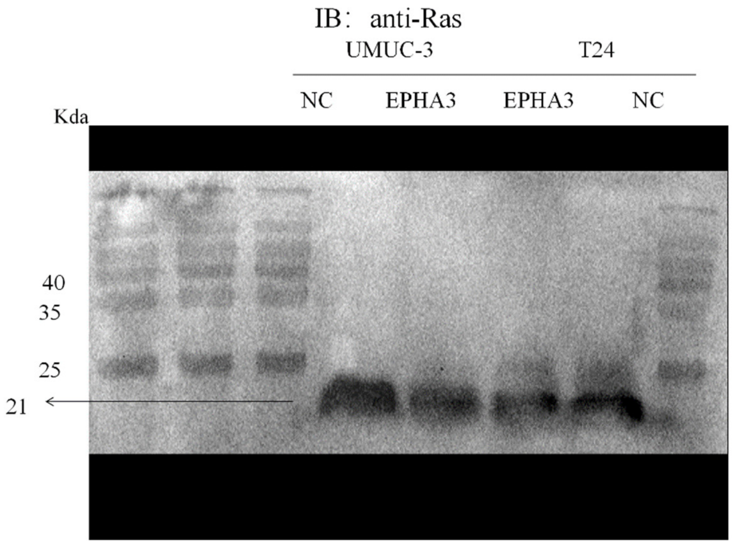

Figure S5. Ras.

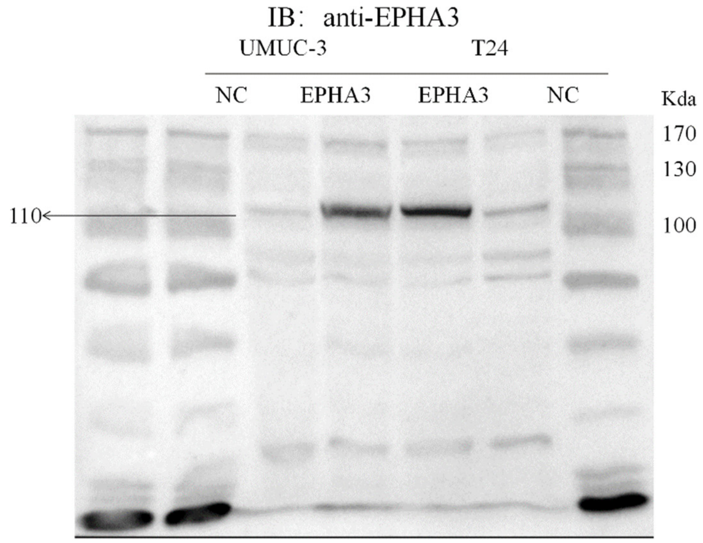

Figure S6. EPHA3.

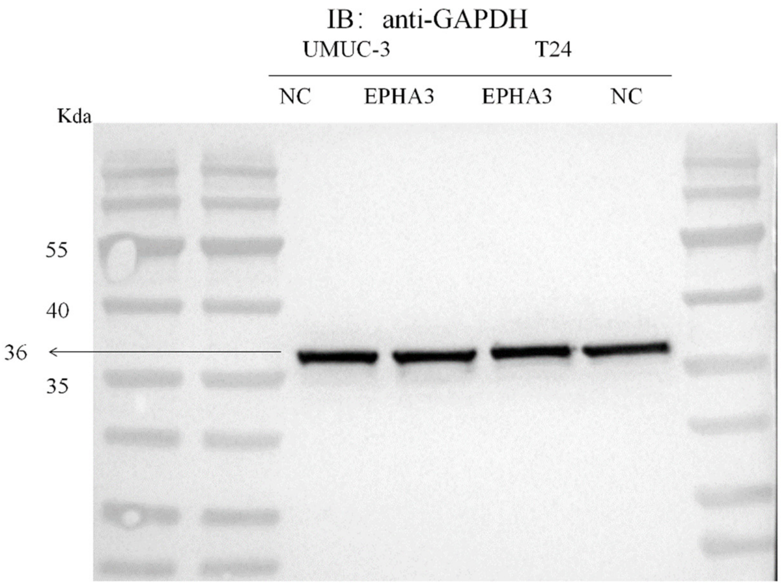

Figure S7. GAPDH.
